# Host interactions of *Aedes albopictus*, an invasive vector of arboviruses, in Virginia, USA

**DOI:** 10.1371/journal.pntd.0009173

**Published:** 2021-02-18

**Authors:** Eliza A. H. Little, Olivia T. Harriott, Karen I. Akaratovic, Jay P. Kiser, Charles F. Abadam, John J. Shepard, Goudarz Molaei

**Affiliations:** 1 Department of Entomology, The Connecticut Agricultural Experiment Station, New Haven, Connecticut, United States of America; 2 Center for Vector Biology & Zoonotic Diseases and Northeast Regional Center for Excellence in Vector-borne Diseases, The Connecticut Agricultural Experiment Station, New Haven, Connecticut, United States of America; 3 Biology Department, Fairfield University, Fairfield, Connecticut, United States of America; 4 Suffolk Mosquito Control, Department of Public Works, Suffolk, Virginia, United States of America; 5 Department of Environmental Sciences, The Connecticut Agricultural Experiment Station, New Haven, Connecticut, United States of America; 6 Department of Epidemiology of Microbial Diseases, Yale School of Public Health, New Haven, Connecticut, United States of America; University of Cincinnati, UNITED STATES

## Abstract

**Background:**

As an invasive mosquito species in the United States, *Aedes albopictus* is a potential vector of arboviruses including dengue, chikungunya, and Zika, and may also be involved in occasional transmission of other arboviruses such as West Nile, Saint Louis encephalitis, eastern equine encephalitis, and La Crosse viruses. *Aedes albopictus* feeds on a wide variety of vertebrate hosts, wild and domestic, as well as humans.

**Methodology/Principal findings:**

In order to investigate blood feeding patterns of *Ae*. *albopictus*, engorged specimens were collected from a variety of habitat types using the Centers for Disease Control and Prevention light traps, Biogents Sentinel 2 traps, and modified Reiter gravid traps in southeast Virginia. Sources of blood meals were determined by the analysis of mitochondrial *cytochrome b* gene sequences amplified in PCR assays. Our aims were to quantify degrees of *Ae*. *albopictus* interactions with vertebrate hosts as sources of blood meals, investigate arboviral infection status, assess the influence of key socioecological conditions on spatial variability in blood feeding, and investigate temporal differences in blood feeding by season. Analysis of 961 engorged specimens of *Ae*. *albopictus* sampled between 2017–2019 indicated that 96%, 4%, and less than 1% obtained blood meals from mammalian, reptilian, and avian hosts, respectively. Domestic cats were the most frequently identified (50.5%) hosts followed by Virginia opossums (17.1%), white-tailed deer (12.2%), and humans (7.3%), together representing 87.1% of all identified blood hosts. We found spatial patterns in blood feeding linked to socioecological conditions and seasonal shifts in *Ae*. *albopictus* blood feeding with implications for understanding human biting and disease risk. In Suffolk Virginia in areas of lower human development, the likelihood of human blood feeding increased as median household income increased and human blood feeding was more likely early in the season (May-June) compared to later (July-October). Screening of the head and thorax of engorged *Ae*. *albopictus* mosquitoes by cell culture and RT-PCR resulted in a single isolate of Potosi virus.

**Conclusion and significance:**

Understanding mosquito-host interactions in nature is vital for evaluating vectorial capacity of mosquitoes. These interactions with competent reservoir hosts support transmission, maintenance, and amplification of zoonotic agents of human diseases. Results of our study in conjunction with abundance in urban/suburban settings, virus isolation from field-collected mosquitoes, and vector competence of *Ae*. *albopictus*, highlight the potential involvement of this species in the transmission of a number of arboviruses such as dengue, chikungunya, and Zika to humans. Limited interaction with avian hosts suggests that *Ae*. *albopictus* is unlikely to serve as a bridge vector of arboviruses such as West Nile and eastern equine encephalitis in the study region, but that possibility cannot be entirely ruled out.

## Author summary

Native to Southeast Asia, breeding populations of *Aedes albopictus* were first discovered in Harris County, Texas, in 1985, and as of 2017, seasonal populations of this species have been reported in more than 40 states and the District of Columbia. *Aedes albopictus* breed readily in natural or man-made environment where stagnant water can accumulate such as gutters, flowerpots, discarded tires, and tree holes. This mosquito species has been implicated in outbreaks of chikungunya, dengue, and Zika viruses and is a competent vector of many arboviruses including West Nile, eastern equine encephalitis, yellow fever, Rift Valley fever, and Japanese encephalitis. *Aedes albopictus* is regarded as an opportunistic mosquito feeding on a variety of domestic and wild mammals, birds, reptiles and amphibians; however, a preference for human blood meals has been noted in blood meal analysis of field-collected mosquitoes and in laboratory investigations. We studied vector-host interactions of *Ae*. *albopictus* in Virginia, United States using molecular methods and identified ten mammalian, three reptilian, and two avian species as blood hosts of this mosquito species. Our study clarifies the host associations of *Ae*. *albopictus* and highlights concerns about the potential role of this mosquito species in transmission of emerging and reemerging arboviruses.

## Introduction

The invasion and spread of *Aedes albopictus* (Skuse, 1894), in the United States have likely occurred since 1985 [1,2]. In its native range, *Ae*. *albopictus* inhabits forests and forest edges, developing in tree holes and other small natural reservoirs [3]. Its domestication and ability to use peridomestic artificial containers, especially tires, enabled its global spread on the heels of human movement and trade [4]. As its range continues to expand, *Ae*. *albopictus* appears to be more closely associated with humans [2,5] and in these areas may even preferentially bite humans [6]. However, *Ae*. *albopictus* inhabit a wide range of environments, from urban to rural, and bite a wide variety of mammalian hosts including humans, domestic and wild animals, reptiles, birds and amphibians [7–9].

*Aedes albopictus* is a vector for viral pathogens causing human diseases including dengue (DENV), chikungunya (CHIKV), and Zika (ZIKV), and it is implicated in outbreaks of these diseases in recent years [10–17]. Both CHIKV and ZIKV have adapted to *Ae*. *albopictus* in areas where their primary vector, *Aedes aegypti*, is absent or outnumbered by *Ae*. *albopictus* [15]. In an investigation of CHIKV outbreak in Italy, *Ae*. *albopictus* was the only mosquito species that tested positive for the virus [18]. Additionally, *Ae*. *albopictus* may vector other arboviruses including but not limited to West Nile virus (WNV), Saint Louis encephalitis virus (SLEV), eastern equine encephalitis virus (EEEV), La Crosse virus (LACV) [19], and dirofilarial worms [4,10]. The role of this species as a vector of arboviruses has yet to be fully elucidated especially in areas where it has been introduced, such as Europe and North America [10,20,21].

In the United States, *Ae*. *albopictus* readily bite mammals, including humans [8,22–26]. Notoriously, *Ae*. *albopictus* is a nuisance biter, supporting its proclivity for human blood when available [27,28]. *Aedes albopictus* will also readily feed on other vertebrate species [20,29] in the absence of its preferred human hosts [30,31]. With its potential for generalist host feeding [3,32] and broad viral susceptibility [10], *Ae*. *albopictus* may be an important vector of arboviruses when underlying conditions are met. Variation in *Ae*. *albopictus* blood feeding may in part be explained by underlying and interrelated factors—host availability and environmental conditions. Human development (e.g. rural vs. urban) [8,33,34] and median household income [26] are conditions linked to spatial variability in *Ae*. *albopictus* blood feeding. Here we aim to ascertain if these two factors explain variability in blood feeding of this mosquito species in Suffolk, Virginia.

As *Ae*. *albopictus* continues to expand its range in North America [2,35], it is important to assess the role of this mosquito as a vector of arboviruses especially in populous areas of high human disease potential. Thus, for the current study engorged mosquitoes were collected from a variety of habitats in Suffolk, Virginia, from 2017 through 2019, and sources of blood meals were determined by the analysis of mitochondrial *cytochrome b* gene sequences amplified in PCR assays. Our objectives were to 1) quantify degrees of *Ae*. *albopictus* interactions with various vertebrate hosts as sources of blood meals and investigate the status of infection with arboviruses, 2) assess the influence of key socioecological conditions, human development and median household income (MHI) on spatial variability in *Ae*. *albopictus* host feeding, and 3) investigate temporal differences in host feeding by season.

## Methods

### Ethics statement

All 50 field sites are located on parcels owned by the city of Suffolk, Virginia or where privately-owned, permissions from the landowner were given. As some of the coauthors are employed by the city of Suffolk Public Works Department, they were authorized to evaluate, collect and control mosquitoes at these locations, and no further permissions were required. No endangered or protected species were involved throughout the field studies for this project.

### Study area

**Fig 1:**
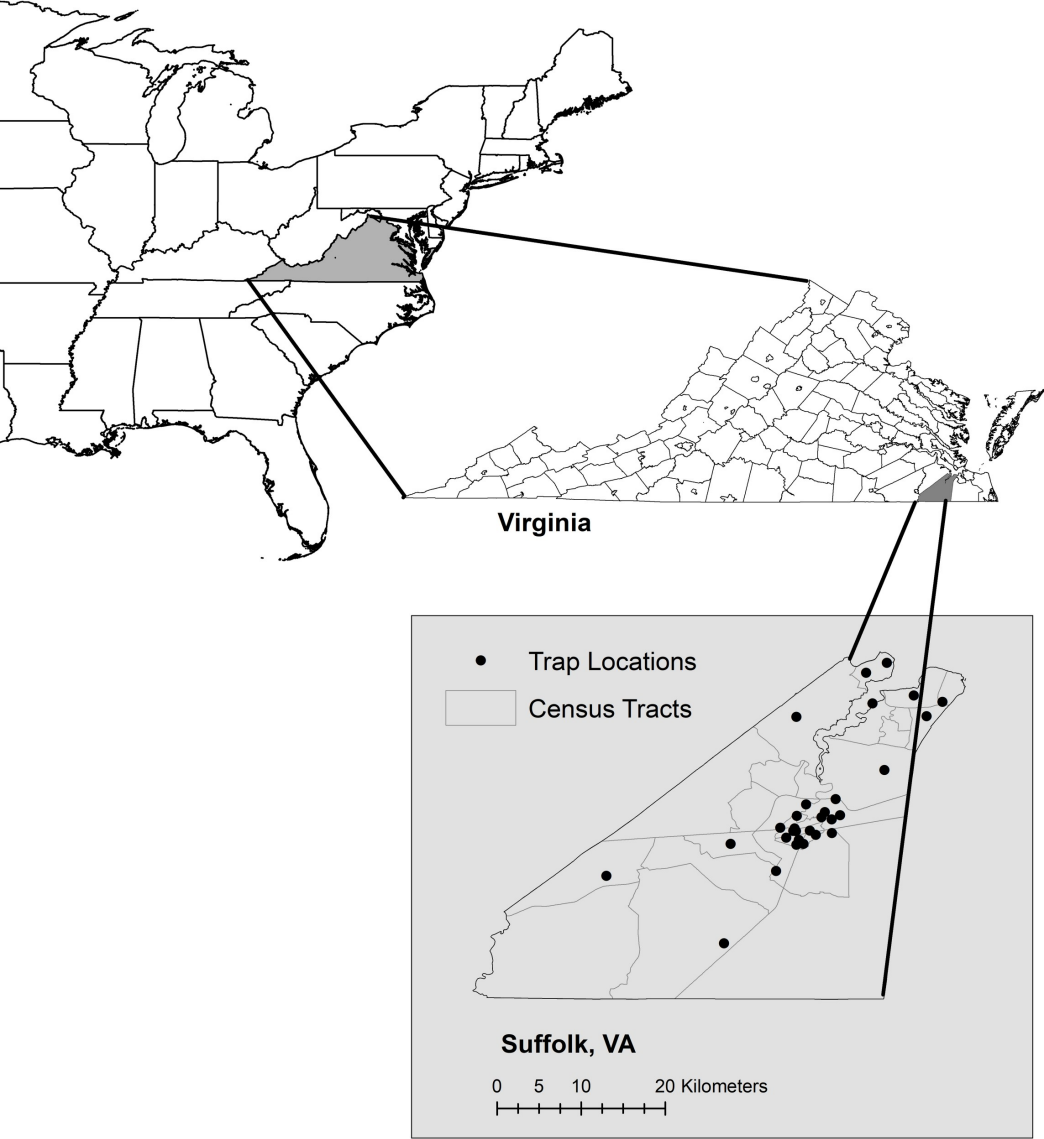
Map of the study area. The city of Suffolk is located in the southeastern corner of the State of Virginia.

The city of Suffolk, Virginia (36°44′ 29″ N 76° 36′ 36″ W) is located in the southeastern corner of the state in the Hampton Roads, Tidewater area, between the upland and lowland coastal plain provinces (Fig 1). The Chesapeake Bay lies 15 km to the north, the Atlantic Ocean is 50 km to the east, and the city hosts three watersheds–the Chowan River watershed in the southwest corner, Great Dismal Swamp watershed in the east, and James River watershed in the north. Elevation ranges from sea level to 33.5 m at the highest point. The city is the largest in the state by land area with about 1,036 km^2^ and in contrast has the second lowest human population density of Virginia’s independent cities with only about 85,000 residents. Land is zoned as agriculture (59%); mixed urban, suburban, and commercial (26%); and conservation (15%). The conservation area consists of two national wildlife refuges. The Nansemond National Wildlife Refuge is in the northern section of the city with over 1.5 km^2^ of salt marsh, grassland, and forested stream habitats. The Great Dismal Swamp National Wildlife Refuge along the eastern edge of the city encompasses over 453 km^2^ of freshwater hardwood swamp habitat, 148 km^2^ of which are within Suffolk borders.

### Mosquito collection

Citywide mosquito control surveillance took place with a variety of traps set annually from April to November at 50 sites throughout the city on a weekly schedule. Centers for Disease Control and Prevention (CDC) light traps, Biogents (BG) Sentinel 2 traps, and modified Reiter gravid traps were set most commonly at 32, 19, and 11 traps per week, respectively. The CDC light traps were most useful and effective along or within wooded areas in Suffolk, VA where they collect primarily *Culiseta melanura*. During the study period as well as the 10-year average, the CDC light traps collected >70% of the *Cs*. *melanura*. Depending on habitat, environmental conditions, and season, CDC light traps were also very effective for collecting temporary floodwater and woodland pool mosquitoes such as *Aedes atlanticus* (68%), *Aedes canadensis* (63%), *Aedes vexans* (64%), and *Psorophora columbiae* (65%), when compared to the other trap types. However, these traps failed to attract *Ae*. *albopictus*, collecting on average less than 2% of this species throughout the city. The BG-Sentinel 2 traps were very effective for collecting *Ae*. *albopictus* and were typically set in urban and suburban areas where this species is most prolific. During the study period as well as the 10-year average, BG-Sentinel traps collected over 97% of the *Ae*. *albopictus*. In many urban and suburban neighborhoods bordering the Great Dismal Swamp National Wildlife Refuge as well as in other heavily wooded areas, BG-Sentinel traps were very efficient at collecting other mosquito species including *Aedes triseriatus* (81%), *Psorophora ferox* (82%), and *Cs*. *melanura* (25%). Finally, the Reiter gravid traps with organic water infusion as lure targeted primarily *Culex pipiens* and *Culex restuans*. These gravid traps collected over 86% of the two *Culex* species throughout the city.

Traps were set in the afternoons, between 1100–1500 h and picked up the following morning between 0700–0900 h. Chambers with live mosquitoes were brought back to the Suffolk Mosquito Control laboratory where they were immobilized using a standard chest freezer set to -25°C. Specimens were transferred to glass Petri dishes and morphologically identified to species using the most recent identification guide for the mid-Atlantic Region [36]; *Ae*. *albopictus* with visible blood meals were individually vialed and shipped on dry ice to the Connecticut Agricultural Experiment Station for host-blood meal analyses and virus testing.

### Blood meal analysis: Genomic DNA extraction and PCR amplification of mitochondrial *cytochrome b* gene sequences

Blood-fed *Ae*. *albopictus* mosquitoes stored in microcentrifuge tubes at -80°C were placed on dry ice before dissection. Each mosquito was placed onto a chilled clean microscope slide, and abdomens were removed under a dissecting scope with a sterile disposable pipette tip. Genomic DNA from the abdomens was extracted using the DNeasy Blood & Tissue Kit protocol (Qiagen, Valencia, CA) with an added homogenization step to enhance lysis. Extracted genomic DNA was used as a template in the polymerase chain reaction using primers specific for avian and mammalian mitochondrial *cytochrome b* gene sequences as previously described [37–39]. Primer pairs to amplify avian *cytochrome b* gene sequences were 5’-GACTGTGACAAAATCCCNTTCCA-3’ (forward) and 5’-GGTCTTCATCTYHGGYTTACAAGAC-3’ (reverse), with an amplified product size of 508 bp. Primer pairs to amplify mammalian *cytochrome b* gene sequences were 5’-CGAAGCTTGATATGAAAAACCATCGTTG-3’ (forward) and 5’-TGTAGTTRTCWGGGTCHCCTA-3’ (reverse), with an amplified product size of 772 bp. Amplicons were analyzed on a 1.3% agarose gel to confirm product size and purified using the QIAquick PCR Purification Kit (Qiagen). Sanger sequencing of purified amplicons in the forward and reverse directions was performed on a 3730xl DNA Analyzer (Applied Biosystems, Foster City, CA) at the Keck Sequencing Facility (Yale University, New Haven, CT). Sequences of both strands were annotated using ChromasPro version 1.7.5 (Technelysium Pty Ltd., Tewantin, Australia) and compared to publicly available sequences in GenBank using the National Center for Biotechnology Information (NCBI) BLASTn search tool (https://blast.ncbi.nlm.nih.gov/Blast.cgi?PROGRAM=blastn&PAGE_TYPE=BlastSearch&LINK_LOC=blasthome). A positive identification was made when >97% identity was attained between the query and subject sequence. A subsample of the resulting annotated sequences was deposited into the NCBI GenBank (Accession numbers: MW267826, MW267827, MW267828, MW267829, MW291654, MW291655, MW291656, MW291657, MW291658, MW291659, MW291660, MW316478, MW323414, MW323415, MW323416, MW323417, MW323418, MW323419, MW323420, MW323421, MW323422, MW323423, MW323424, MW323425).

Although the methodology used for blood meal analysis in this study has been previously used with much success in numerous investigations, 22.4% (n = 277) of the total 1,238 slightly, partially, or fully engorged *Ae*. *albopictus* did not meet the criteria to assign a host species and were assumed unknown. These could be due to the amount of blood acquired by *Ae*. *albopictus* mosquitoes, the time between capturing mosquitoes and processing for blood meal analysis, quality of the isolated DNA, availability of the species-specific *cytochrome b* gene sequences in the database, and the degrees of sequence homology among vertebrate hosts in the study area. Furthermore, co-amplification of *Ae*. *albopictus* with vertebrate host DNA in blood meal analysis has been reported and could be due to the matching sequences in mosquito and vertebrate host genomes and primers used [25].

### Analysis of socioecological characteristics and *Ae*. *albopictus* blood feeding

**Fig 2:**
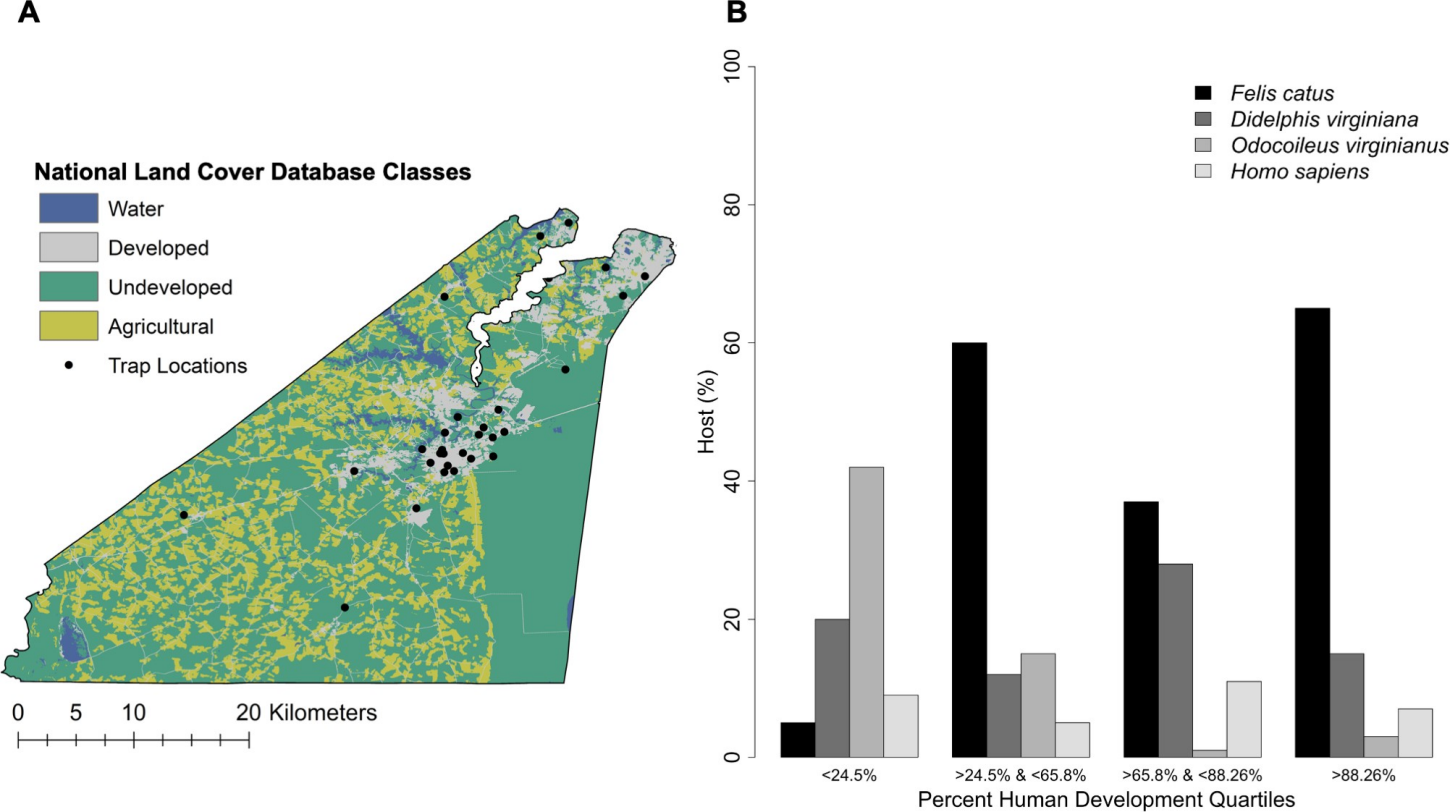
Human Development in Suffolk. (A) National Land Cover Database classification for open water, developed, undeveloped, and agricultural land in Suffolk. (B) The proportion of blood meals from domestic cats (*Felis catus*), Virginia opossums (*Didelphis virginiana*), white-tailed deer (*Odocoileus virginianus*), and humans (*Homo sapiens*) across quartiles of human development.

**Fig 3:**
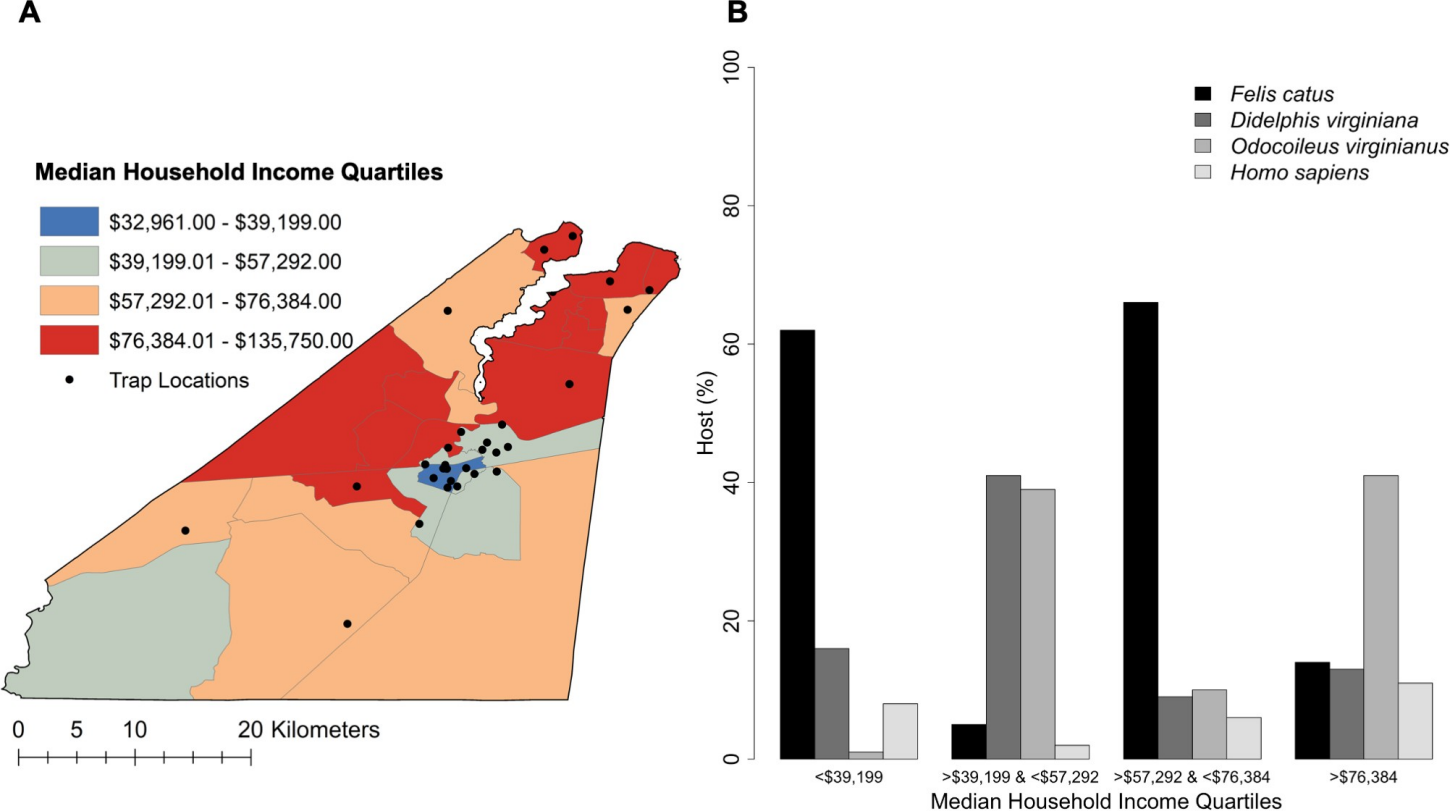
Median Household Income in Suffolk. (A) Median household income classified by quartile in Suffolk. (B) The proportion of blood meals from domestic cat (*Felis catus*), Virginia opossum (*Didelphis virginiana*), white-tailed deer (*Odocoileus virginianus*), and human (*Homo sapiens*) across quartiles of median household income.

A geographic information system (GIS) model was created so that the spatial patterns of blood-feeding activity in relation to socioecological characteristics (human development and MHI) could be explored and evaluated. ArcGIS version 10.8 (Esri, Redlands, California) was used for mapping and analyses. The 2016 National Land Cover Database breaks land cover into 20 classes at a spatial scale of 30 m; however, a simplified classification was used to distinguish between open water, developed, undeveloped, and agricultural land cover classes (Fig 2A). Because *Ae*. *albopictus* has a flight range of under 200 m [40–42], we calculated the proportion of developed land within 200 m of each trap location. We used this metric of human development because it provides a more location-specific measurement of human development than population density which can be calculated by census tract. For each census tract in the city of Suffolk, we accessed the 2018 MHI (US Census Bureau 2018, Table B19013) (Fig 3A). Logistic models (family = binomial) were used to assess the influence of socioecological characteristics (human developed and MHI) and their interaction on host feeding differences. For the regression analyses, we standardized the explanatory variables by subtracting the mean and dividing by the standard deviation. Using standardized explanatory variables allows us to interpret the results more easily, the effect change (“each unit change”) of an explanatory variable on the likelihood of *Ae*. *albopictus* host interaction is measured in standard deviations. Finally, we included a three-category variable for season (early–May and June; mid–July and August; and late–September and October) in the model to assess temporal shifts in *Ae*. *albopictus* blood feeding. All statistical analyses were completed using R Statistical Software version 3.6.2.

### Virus isolation and identification

The head and thorax of each blood-fed *Ae*. *albopictus* were homogenized in 1 mL phosphate-buffered saline containing 30% heat-inactivated rabbit serum, 0.5% gelatin, and 1× antibiotic/antimycotic by using a copper BB and vibration mill as previously described [43]. Mosquito homogenates were centrifuged at 4°C for 10 minutes at 520g, and 100 μL of the supernatant was inoculated onto a monolayer of confluent Vero cells growing in minimal essential media, 5% fetal bovine serum, and 1× antibiotic/antimycotic. Cells were maintained at 37°C in 5% CO_2_ and examined daily for cytopathic effect from Day 3 through Day 7 after inoculation. RNA from infected cell supernatants was extracted by using a viral RNA Mini Kit (Qiagen, Valencia, CA) and screened in real-time reverse transcriptase-polymerase chain reaction (RT-PCR) assays using the TaqMan RT-PCR Ready-Mix Kit (Applied Biosystems) for CHIKV [44], WNV [45], and ZIKAV [46].

When the aforementioned viruses were not detected, the RNA was amplified using the Titan One-Tube RT-PCR System (Roche Diagnostics, Indianapolis, IN) and universal bunyavirus primers BUNS^+^new: 5’-TGACCAGTAGTGTACTCCAC-3’ and BUNS^–^new: 5’-CAAGCAGTAGTGTGCTCCAC-3’, as previously described [47,48]. The amplified product was then purified using the QIAquick PCR Purification Kit (Qiagen) and sequenced at the Yale DNA sequencing facility (New Haven, CT) using a 3730xl 96-capillary genetic analyzer (Applied Biosystems). Sequences were annotated using ChromasPro software (Technelysium) and identified through a BLAST search of the GenBank database.

## Results

A total of 384,243 female mosquitoes in 34 species were collected at 30 trap sites during 3,038 trap nights (1,436 BG-Sentinel traps, 597 CDC miniature light traps, and 1,005 modified Reiter gravid traps) between 2017–2019. The most frequently-collected species were *Cs*. *melanura* and *Ae*. *albopictus*, comprising 43.0% (n = 165,124) and 23.6% (n = 90,628) of the overall collection, respectively (Table 1).

**Table 1:** Number and percentage of adult female mosquitoes collected from sites where blood fed *Ae*. *albopictus* were collected in Suffolk, Virginia, 2017 to 2019.

Mosquito Species	No.	%
*Culiseta melanura*	165,124	43.0%
*Aedes albopictus*	90,628	23.6%
*Culex pipiens/restuans*	30,578	8.0%
*Psorophora ferox*	24,906	6.5%
*Aedes canadensis*	16,830	4.4%
*Aedes atlanticus*	12,355	3.2%
*Culex salinarius*	10,396	2.7%
*Coquillettidia perturbans*	7,314	1.9%
*Aedes vexans*	5,166	1.3%
*Culex erraticus*	4,556	1.2%
*Anopheles crucians*	3,287	0.9%
*Anopheles quadrimaculatus*	2,696	0.7%
*Psorophora columbiae*	2,380	0.6%
*Aedes infirmatus*	2,168	0.6%
*Aedes triseriatus*	2,037	0.5%
*Anopheles punctipennis*	1,587	0.4%
*Uranotaenia sapphirina*	835	0.2%
*Aedes tormentor*	503	0.1%
*Culex territans*	277	0.1%
*Aedes japonicus*	188	< 0.1%
*Psorophora ciliata*	55	< 0.1%
*Aedes taeniorhynchus*	41	< 0.1%
*Psorophora howardii*	39	< 0.1%
*Ochlerotatus sollicitans*	38	< 0.1%
*Orthopodomyia signifera*	22	< 0.1%
*Aedes sticticus*	18	< 0.1%
*Toxorhynchites rutilus septentrionalis*	12	< 0.1%
*Psorophora mathesoni*	7	< 0.1%
*Culex nigripalpus*	3	< 0.1%
*Psorophora horrida*	3	< 0.1%
*Aedes dupreei*	2	< 0.1%
*Aedes cantator*	1	< 0.1%
*Aedes mitchellae*	1	< 0.1%
*Aedes trivittatus*	1	< 0.1%
Damaged-Unidentifiable*	189	< 0.1%
Total	384,243	

*Damaged-Unidentifiable indicates specimens that were unidentifiable to species by morphological characteristics due to severe damage from environmental conditions and/or trapping equipment.

**Fig 4:**
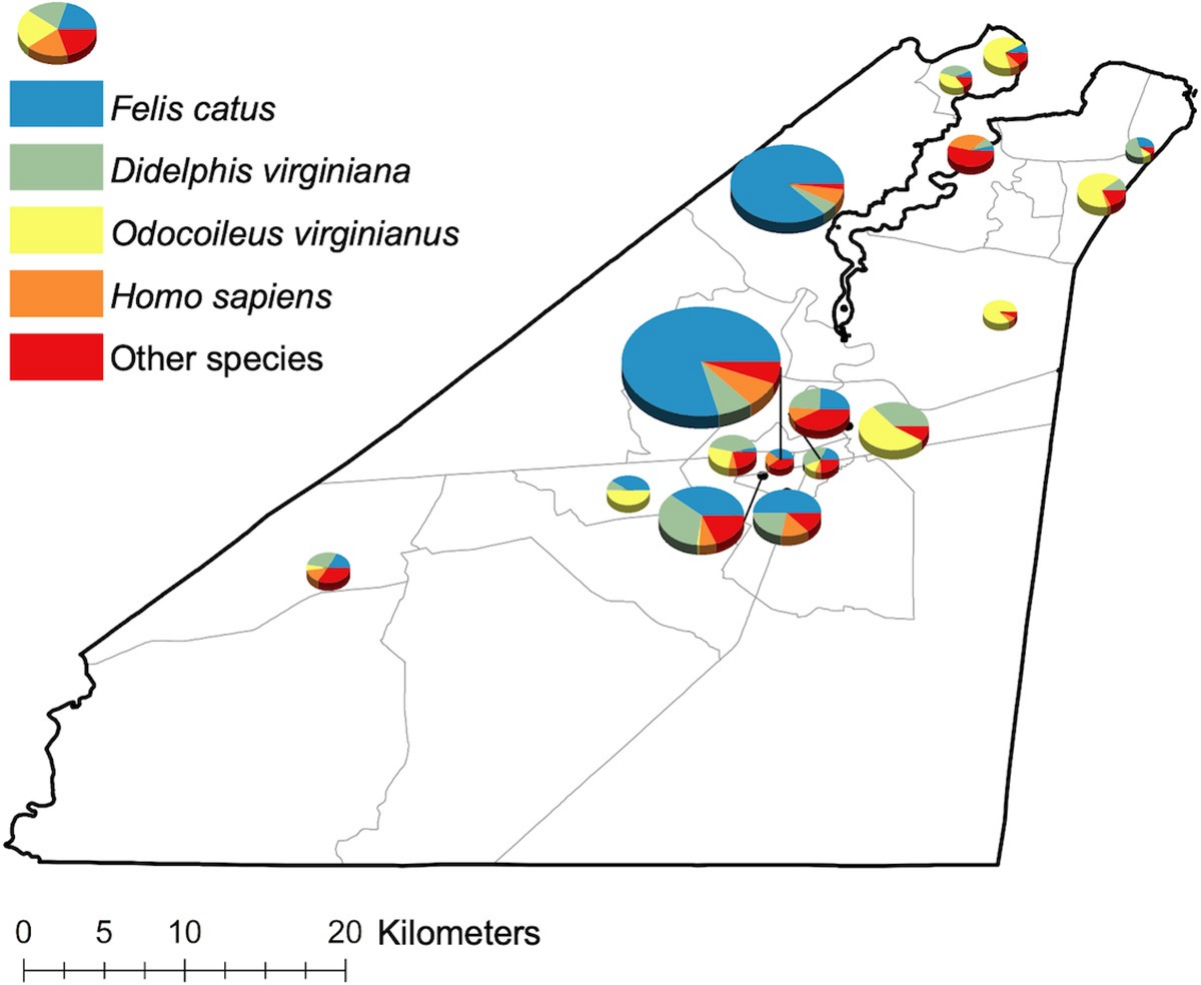
Spatial distribution of *Ae*. *albopictus* blood meals. The proportion of blood meals from domestic cats (*Felis catus*), Virginia opossums (*Didelphis virginiana*), white-tailed deer (*Odocoileus virginianus*), and humans (*Homo sapiens*) across Suffolk. Pie charts only illustrated for trap sites with more than 10 identified *Ae*. *albopictus* blood meals.

A total of 1,238 slightly, partially, or fully engorged *Ae*. *albopictus* collected between 2017 and 2019 were subjected to blood meal analysis. Of these, 77.6% (n = 961) produced conclusive host feeding results. Overall, 95.8% (n = 921) of the *Ae*. *albopictus* fed on mammals, 3.9% (n = 37) on turtles, and 0.3% (n = 3) on birds. Two of the three avian blood meals were mixed with mammalian blood. The four most common hosts were all mammals: domestic cat (50.5%, n = 485), Virginia opossum (17.1%, n = 164), white-tailed deer (12.2%, n = 117), and human (7.3%, n = 70), together representing 87.1% of all identified blood hosts (Table 2; Fig 4).

**Table 2:** Number and percentage of *Ae*. *albopictus* blood meals collected in Suffolk, Virginia, 2017–2019.

Vertebrate Host Common Name (Species Name)	Frequency of Blood Meal No. (%)
Domestic cat (*Felis catus*)	485 (50.47%)
Virginia opossum (*Didelphis virginiana*)	164 (17.07%)
White-tailed deer (*Odocoileus virginianus*)	117 (12.17%)
Human (*Homo sapiens*)	70 (7.28%)
Common box turtle (*Terrapene carolina*)	34 (3.54%)
Black rat (*Rattus rattus*)	28 (2.91%)
Dog (*Canis lupus familiaris)*	22 (2.29%)
Eastern gray squirrel (*Sciurus carolinensis*)	19 (1.98%)
Eastern cottontail rabbit (*Sylvilagus floridanus*)	12 (1.25%)
Raccoon (*Procyon lotor*)	2 (0.21%)
Gray fox (*Urocyon cinereoargentus*)	2 (0.21%)
Eastern box turtle (*Terrapene carolina carolina*)	2 (0.21%)
Common musk turtle (*Sternotherus odoratus*)	1 (0.10%)
American robin (*Turdus migratorius*)	1 (0.10%)
Common grackle & white-tailed deer (*Quiscalus quiscula* & *Odocoileus virginianus*)	1 (0.10%)
American robin & Virginia opossum (*Turdus migratorius* & *Didelphis virginiana*)	1 (0.10%)
Total	961

Across trap locations in Suffolk, MHI ranged from $32,961 to $98,011 (Mean = $58,904; SD = $21,037) and human development (the percent of developed land within 200 m of trap locations) ranged from 6.71% to 100% (Mean = 56.66%; SD = 34.18%). Table 3 presents the logistic regression modeling results, odds ratios (OR) and 95% confidence intervals (CI), for domestic cats, Virginia opossums, white-tailed deer, and humans. For each unit increase in human development, the odds that *Ae*. *albopictus* fed on domestic cats increased by 27% (OR = 1.274; 95% CI 1.001–1.616) while the odds that *Ae*. *albopictus* fed on white-tailed deer decreased by 61% (OR = 0.386; 95% CI 0.270–0.537). There were no significant differences in *Ae*. *albopictus* feeding on Virginia opossums (OR = 1.277; 95% CI 0.971–1.678) or humans (OR = 0.996; 95% CI 0.472–1.793) by human development (Table 3; Fig 2B). For each unit increase in MHI, the odds that *Ae*. *albopictus* fed on domestic cats decreased by 22% (OR = 0.781; 95% CI 0.609–0.997), while the odds that *Ae*. *albopictus* fed on white-tailed deer increased by 111% (OR = 2.109; 95% CI 1.472–3.048). There were no significant differences in *Ae*. *albopictus* feeding on Virginia opossums (OR = 0.905; 95% CI 0.641–1.254) or humans (OR = 1.065; 95% CI 0.513–1.844) by MHI (Table 3; Fig 3B).

**Table 3:** Logistic regression results (odds ratios and 95% confidence intervals) for domestic cats, Virginia opossums, white-tailed deer, and humans.

	Domestic cat	Virginia opossum	White-tailed deer	Human
Intercept	0.522 (0.371–0.729)	0.298 (0.194–0.449)	0.019 (0.065–0.205)	0.083 (0.039–0.154)
DEV	1.274 (1.001–1.616)	1.277 (0.971–1.678)	0.386 (0.270–0.537)	0.996 (0.472–1.793)
MHI	0.781 (0.609–0.997)	0.905 (0.641–1.254)	2.109 (1.472–3.048)	1.065 (0.513–1.844)
Mid-Season	1.836 (1.314–2.571)	0.578 (0.356–0.926)	1.337 (0.733–2.507)	0.438 (0.249–0.761)
Late-Season	1.200 (0.843–1.712)	1.528 (0.992–2.381)	1.718 (0.944–3.229)	0.343 (0.168–0.666)
MHI X DEV	0.774 (0.631–0.949)	1.791 (1.330–2.454)	2.496 (1.811–3.526)	0.557 (0.334–0.848)

DEV is human development and MHI is median household income. Mid-Season refers to July and August while Late-Season refers to September and October and the coefficients are in reference to Early season which refers to May and June. MHI X DEV is the interaction between median household income and human development.

**Fig 5:**
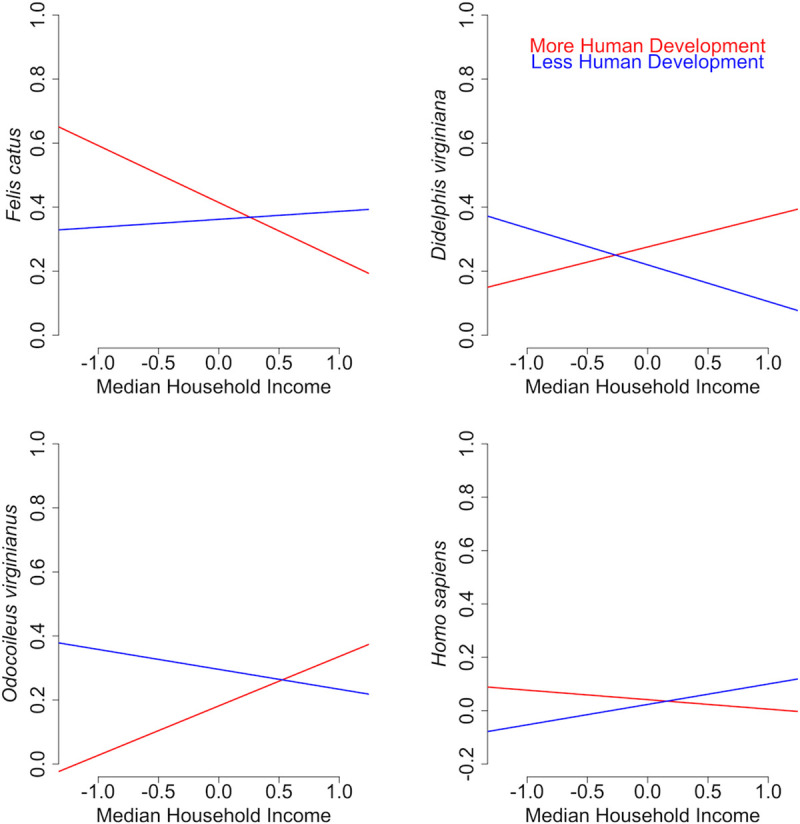
Mediating effects. Mediating effects of human development on the influence of median household income on *Ae*. *albopictus* host interactions with domestic cats (*Felis catus*), Virginia opossums (*Didelphis virginiana*), white-tailed deer (*Odocoileus virginianus*), and humans (*Homo sapiens*).

We modeled the two-way interactions between the socioecological conditions, human development and MHI, and found that domestic cat, Virginia opossum, white-tailed deer and human feeding were all mediated by human development. In areas where human development was high (i.e. above the mean for Suffolk), the likelihood of a blood meal taken from a domestic cat decreased (OR = 0.774; 95% CI 0.631–0.949), while the likelihood of a blood meal acquired from a Virginia opossum (OR = 1.791; 95% CI 1.330–2.454) or white-tailed deer (OR = 2.496; 95% 1.811–3.526) increased as MHI increased. The likelihood of a blood meal taken from a human increased with MHI in areas where human development was low (i.e. below the mean for Suffolk) (OR = 0.557; 95% CI 0.334–0.848) (Table 3; Fig 5).

We found temporal differences in blood feeding by season. More domestic cats were fed upon in July and August compared to May and June (OR = 1.836; 95% CI 1.314–2.571). Less Virginia opossums were fed on in July and August compared to May and June (OR = 0.578; 95% CI 0.356–0.926). Less humans were fed on in July and August (OR = 0.438; 95% CI 0.249–0.761) and in September and October (OR = 0.343; 95% CI 0.168–0.666) compared to May and June suggesting that there is higher *Ae*. *albopictus* biting pressure on people early in the season (May and June) compared to later months (Table 3).

Screening head and thorax of engorged *Ae*. *albopictus* by cell culture resulted in a single viral isolate. The virus isolate was further characterized by RT-PCR using primers that targeted the conserved terminal ends of the S-segment of the *Orthobunyavirus* genus. The identity of the virus was determined as Potosi virus (POTV). The virus-positive specimen had been collected on 12 September 2019 from a suburban residential neighborhood located to the north of Suffolk, VA (36.910420° N, 76.488010° W) and the source of blood meal was a white-tailed deer. Screening for other arboviruses including CHIKV, WNV, and ZIKV did not produce any positive PCR result.

Between 2010–2019, a total of 26,241 pools of 12 mosquito species including *Cs*. *melanura*, *Cx*. *pipiens/restuans*, *Ae*. *albopictus*, and *Culex erraticus* were screened for WNV and EEEV by RT-PCR. Of these, 58 pools of *Cs*. *melanura* and 151 pools of *Cx*. *pipiens/restuans* tested positive for WNV. In addition, 574 pools of *Cs*. *melanura* and one pool of *Cx*. *erraticus* tested positive for EEEV (Table 4).

**Table 4:** Number of mosquito pools tested positive for West Nile and eastern equine encephalitis viruses in Suffolk, Virginia, 2010–2019.

Species of Mosquito	No. of Pools Tested	WNV Positive	EEEV Positive
*Culiseta melanura*	17,373	58	574
*Culex pipiens/restuans*	2,828	151	0
*Aedes albopictus*	2,556	0	0
*Culex salinarius*	1,228	0	0
*Culex erraticus*	1,032	0	1
*Aedes vexans*	568	0	0
*Coquillettidia perturbans*	306	0	0
*Uranotaenia sapphirina*	271	0	0
*Aedes triseriatus*	70	0	0
*Anopheles quadrimaculatus*	4	0	0
*Aedes japonicus*	4	0	0
*Culex territans*	1	0	0
Total	26,241	209	575

## Discussion

In our investigation of vector-host interaction, we found that 96% of *Ae*. *albopictus* blood hosts were mammals in the city of Suffolk, Virginia. Our analysis revealed more blood feeding on domestic cats (50.5%), Virginia opossums (17.1%), and white-tailed deer (12.2%) than humans (7.3%). Mosquito blood feeding studies routinely characterize mammals as the primary hosts for *Ae*. *albopictus* and many investigations have reported that humans represent the majority of hosts [8,24,25,30,33,49,50]. Some studies have reported that human blood represents almost all blood meals sampled. In Thailand, 94% of blood meals were human-derived [51], and in Cameroon 95% of all *Ae*. *albopictus* blood meals contained human blood [52]. In urban areas of Spain and Singapore, human blood represented 100% of *Ae*. *albopictus* blood meals [29,53]. However, in accordance with our study there are other studies that have shown higher rates of blood feeding on nonhuman mammalian species. Blood meal analyses of *Ae*. *albopictus* collected from rural and urban study sites across multiple states (Missouri, Indiana, Illinois, Louisiana, and Florida) found that 19% [22], and 35% of blood meals were from cottontail rabbits [23]. In a study conducted in five predominantly residential neighborhoods in Baltimore, brown rats (*Rattus norvegicus*) represented 72% of blood meals for *Ae*. *albopictus* [26]. It may be that in these locations, alternative mammalian hosts are more abundant and/or accessible than humans.

Our analysis showed that less than 1% of the blood meals were derived from avian hosts suggesting that *Ae*. *albopictus* is unlikely to serve as a bridge vector of arboviruses such as WNV and EEEV in the study region, but that possibility cannot be entirely ruled out. Other studies have also shown that birds do not appear to be a preferred host group for *Ae*. *albopictus* [9,23,24,33,49]. However, a study in China found that in forested areas with minimal to no human presence, avian blood (32%) was detected almost as frequently as human blood (37%) [11]. In urban areas with many humans, avian blood has also been identified. In Missouri, 21% of blood meals [22] and in Seoul, Korea 26% of blood meals for *Ae*. *albopictus* were from birds [50]. A study on *Ae*. *albopictus* blood feeding that did not find evidence of avian blood feeding concluded that risk of this mosquito species in transmission of avian zoonoses (e.g., WNV, EEEV, SLEV) is minimal [8].

In our analysis, we found that 3.9% (n = 37) of all blood meals were from two species of freshwater turtles. Several other studies have also found low levels (1–2%) of turtle-derived blood meals in *Ae*. *albopictus* [23,24,29,49]. However, one study, in China, reported an even greater propensity of blood feeding (23%) from turtles [11]. As sources of blood meals for various mosquito species, reptiles have been implicated in the transmission cycle of arboviruses, and it has been suggested that these ectothermic vertebrates may substantially influence transmission dynamics as amplification or dilution hosts [54–57]. In an experimental infection of two turtle species collected in southern New England, the spotted turtle, *Clemmys guttata*, developed viremia and neutralizing antibody to 3 logs or more of EEEV. Viremia was not detected in the eastern painted turtle, *Chrysemys picta*, but neutralizing antibodies were detected in one of 15 inoculated animals. Based on these findings, high virus titer and duration of viremia, it was concluded that *C*. *picta* may be involved as overwintering hosts of the EEEV [58]. Eastern and western equine encephalitis viruses have been isolated from snapping, painted, and box turtles in New Jersey, and neutralizing antibodies to EEEV have also been reported in turtles tested [59].

While there is consensus that *Ae*. *albopictus* feed primarily on mammals, local differences in blood feeding analyses results have prompted researchers to investigate differences in host feeding patterns of this mosquito that might be related to other factors such as local environmental conditions and/or host availability and abundance [20]. In our study, we did not find evidence that human blood feeding differed based on human development or MHI. However, we found that the proportion of domestic cat-derived blood meals decreased while the proportion of white-tailed deer-derived blood meals increased with MHI. We also found a clear preponderance of blood meal derived from white-tailed deer in less developed locations and more domestic cat feeding in more developed areas. Finally, we found interactive effects between human development and MHI on host feeding such that in areas where human development was high, the likelihood of feeding on domestic cats decreased, while the likelihood of feeding on Virginia opossums or white-tailed deer increased as MHI increased. The likelihood of a blood meal acquired from a human increased with MHI in areas where human development was below the mean suggesting that people who live in areas of low development and high MHI may be particularly at risk to *Ae*. *albopictus* biting in Suffolk, Virginia. A study based in Italy found a significant difference in the percent of human-derived blood meals between urban (68–91%) and rural sites (18–21%) [34]. Another study in India showed that *Ae*. *albopictus* blood feeding from human hosts was highest in densely built urban areas and progressively decreased as vegetation increased [33]. However, a study in New Jersey found significantly more human blood in suburban (62%) compared to urban areas (43%), while more domestic cat-derived blood meals were identified in urban (28%) compared to suburban (13%) areas [8]. In Baltimore, differences in human blood feeding were noted across neighborhood socioeconomic status with a higher proportion in the neighborhoods defined as lower socioeconomic status [26].

Most studies have focused on vector-host interactions and blood feeding behavior of *Ae*. *albopictus* in urban and suburban settings [9] versus rural environments where humans may be less available [23,34]. Some studies suggest that *Ae*. *albopictus* feed opportunistically on locally abundant and available hosts [23,24]. In a study in the La Réunion Island, it was found that while *Ae*. *albopictus* displayed opportunistic feeding potential, it also preferred humans over other animal hosts [30]. Other studies have also reported that while capable of biting a wide range of animals, *Ae*. *albopictus* primarily feeds on human hosts regardless of local environmental conditions (e.g., host availability and abundance) [29,33,50].

Abundance of non-human hosts could be zooprophylactic by diverting feeding of *Ae*. *albopictus* from human hosts, a possibility that merits further study. In Baltimore, 72% of blood meals were obtained from brown rats, prompting the study authors to question a potential function of the brown rat as a pathogen reservoir that diverts bites from humans and/or a host that contributes to increases in *Ae*. *albopictus* populations [26]. In our study, we found that 50.5% of *Ae*. *albopictus* blood meals were acquired from domestic cats, and similar questions could be raised regarding the role of this felid species. We also found that *Ae*. *albopictus* fed disproportionately on domestic cats in more urban areas, so altering the availability of host species may change *Ae*. *albopictus* host interactions and human biting pressure depending on local environmental conditions. In the city of Suffolk, domestic cats are abundant in the urban areas where engorged *Ae*. *albopictus* were collected. The populations of feral cats in low-income urban areas are unregulated by the city of Suffolk animal control agency, apart from the limited use of animal traps that are rarely used. These cats are free-ranging and nocturnally active, and their behavior of resting in shaded areas of vegetation during the day, where *Ae*. *albopictus* also is active, may contribute to higher preponderance of blood meals form cats. Also, in these urban areas where feral cats are prolific, it is likely that they prey heavily on the resident avian species, thus, possibly impacting the host selection for *Ae*. *albopictus* [60].

White-tailed deer constituted 12.2% of blood meals in *Ae*. *albopictus* in our study. In the city of Suffolk, white-tailed deer frequently inhabit areas of the city where income levels are medium to high and where environmental landscape is more suburban to rural and interspersed with heavily wooded areas. Over the past few decades, the white-tailed deer populations have been closely monitored by the Virginia Department of Wildlife Resources to maintain populations in the city of Suffolk and neighboring jurisdictions in the southeastern Virginia [61]. Strategies to decrease and stabilize the population have been implemented during the past two decades after restorative efforts over the past century led to a significant rebound of the white-tailed deer populations [61].

One specimen of *Ae*. *albopictus* tested positive for POTV in the present study. The source of the blood meal was a white-tailed deer. As a member of the *Orthobunyavirus* (family Bunyaviridae), POTV is maintained in a cycle involving mosquito vectors and deer hosts [62]. Potosi virus has been isolated from field-collected mosquitoes including *Ae*. *albopictus* [63] and in laboratory analysis, this mosquito species has been shown to be a competent vector for this virus [64]. Frequent exposure of white-tailed deer to POTV has also been documented [65,66]. Despite widespread distribution of POTV in the U.S. and recurring exposure of humans resulting in occasional meningitis or encephalitis, the public health significance of this virus is not clearly understood [62].

Except for the single POTV-positive specimen, no other *Ae*. *albopictus* specimen tested positive for any arbovirus including bunyaviruses in our study. However, infection of *Ae*. *albopictus* with LACV has been documented in Virginia [67], Tennessee [68,69], and Texas [70], and human cases of this virus have been reported from Virginia (n = 9) and neighboring states including Kentucky (n = 2), Maryland (n = 3), North Carolina (n = 179), Tennessee (n = 115), and West Virginia (n = 86) over the past 10 years [https://www.cdc.gov/lac/tech/epi.html]. Human cases of Jamestown Canyon virus (JCV) have also been reported from the neighboring states including New Jersey (n = 1), North Carolina (n = 1), and Tennessee (n = 2) [https://www.cdc.gov/jamestown-canyon/statistics/index.html]. These findings suggest the potential contribution of *Ae*. *albopictus* in the spread of the two aforementioned diseases.

## Conclusion

*Aedes albopictus* has successfully invaded and established a global distribution, including in the United States, due in part to human-mediated introduction events, strong interspecific competitive ability [71], ecological plasticity, and challenges associated with population control. The association of *Ae*. *albopictus* with a wide range of mammalian hosts, including humans in the current study, parallels the results of previous examinations of the host feeding behavior of this mosquito as a predominately mammalophagic species in the mid-Atlantic region of the United States. Our findings, in conjunction with abundance in urban/suburban settings, virus isolations from field-collected mosquitoes [68,72], and vector competence of *Ae*. *albopictus*, highlight the potential of this species to transmit several arboviruses such as DENV, CHIKV, ZIKAV, LACV, and JCV to humans. A small percentage of avian-derived blood meals was detected in our study and justifies closer surveillance of *Ae*. *albopictus* populations in Suffolk, Virginia to more definitively determine the potential of this invasive species to serve as an epidemic-epizootic bridge vector in transmission of arboviruses, such as WNV and EEEV, to humans and other mammals.
